# Exploiting a Phage-Bacterium Interaction System as a Molecular Switch to Decipher Macromolecular Interactions in the Living Cell

**DOI:** 10.3390/v10040168

**Published:** 2018-04-01

**Authors:** Éva Viola Surányi, Rita Hírmondó, Kinga Nyíri, Szilvia Tarjányi, Bianka Kőhegyi, Judit Tóth, Beáta G. Vértessy

**Affiliations:** 1 Department of Applied Biotechnology and Food Sciences, Budapest University of Technology and Economics, H-1111 Budapest, Hungary; nyiri.kinga@ttk.mta.hu (K.N.); bianka.kohegyi@gmail.com (B.K.); 2 Institute of Enzymology, RCNS, Hungarian Academy of Sciences, H-1111 Budapest, Hungary; kicsiszilvi.tarjanyi@gmail.com (S.T.); toth.judit@ttk.mta.hu (J.T.)

**Keywords:** gene expression regulation, molecular probe, macromolecular interactions, phage-host interaction

## Abstract

Pathogenicity islands of *Staphylococcus aureus* are under the strong control of helper phages, where regulation is communicated at the gene expression level via a family of specific repressor proteins. The repressor proteins are crucial to phage-host interactions and, based on their protein characteristics, may also be exploited as versatile molecular tools. The Stl repressor from this protein family has been recently investigated and although the binding site of Stl on DNA was recently discovered, there is a lack of knowledge on the specific protein segments involved in this interaction. Here, we develop a generally applicable system to reveal the mechanism of the interaction between Stl and its cognate DNA within the cellular environment. Our unbiased approach combines random mutagenesis with high-throughput analysis based on the *lac* operon to create a well-characterized gene expression system. Our results clearly indicate that, in addition to a previously implicated helix-turn-helix segment, other protein moieties also play decisive roles in the DNA binding capability of Stl. Structural model-based investigations provided a detailed understanding of Stl:DNA complex formation. The robustness and reliability of our novel test system were confirmed by several mutated Stl constructs, as well as by demonstrating the interaction between Stl and dUTPase from the Staphylococcal ϕ11 phage. Our system may be applied to high-throughput studies of protein:DNA and protein:protein interactions.

## 1. Introduction

*Staphylococcus aureus* (*S. aureus*) pathogenicity islands (SaPIs) operate in the fine-tuned control mechanism between phages and the host bacterial cell. SaPIs play an important role in spreading virulence factors [1]. These mobile genetic elements usually encode toxins, antibiotic resistance genes, and antigens responsible for the pathogenicity of the given strain. Pathogenicity islands are considered to be similar to phages since these mobile elements can also move via horizontal transfer from one strain to the other, hijacking the phage capsid. Under normal circumstances, the pathogenicity island is under the repression of a transcription regulatory factor encoded by the island. The master repressor of a specific SaPI, namely SaPI_bov1_, is the Stl protein. Stl binding to a specific DNA segment within SaPI [2,3] prevents the excision and replication of the mobile genetic elements. SaPIs can be mobilized by infecting the bacteria with certain staphylococcal bacteriophages (e.g., Φ11 and 80α phages) or by the induction of endogenous prophages [4]. Following bacteriophage infection, the excision and replication of SaPIs is induced by the formation of a repressor:derepressor complex constituting the master repressor and another phage-related protein. As a result, the repression is terminated and SaPI becomes mobilized, i.e., the SaPI DNA is replicated and packaged into parallel maturing phage particles in the bacterium [5]. In recent years, more and more of these repressor:derepressor interactions have been discovered. Firstly, dUTPases from Φ11 and 80α phages were identified as specific derepressors of Stl function. It has also been shown that Φ11 dUTPase has a stronger effect on the mobilization of SaPI_bov1_ as compared to 80α dUTPase [4]. Interestingly, in addition to the Φ11 and 80α phage dUTPases, both belong to the trimeric dUTPase enzyme family, structurally very different dimeric dUTPases (encoded by different phages) are shown to be also able to derepress the same SaPI island [6,7].

**Figure 1:**
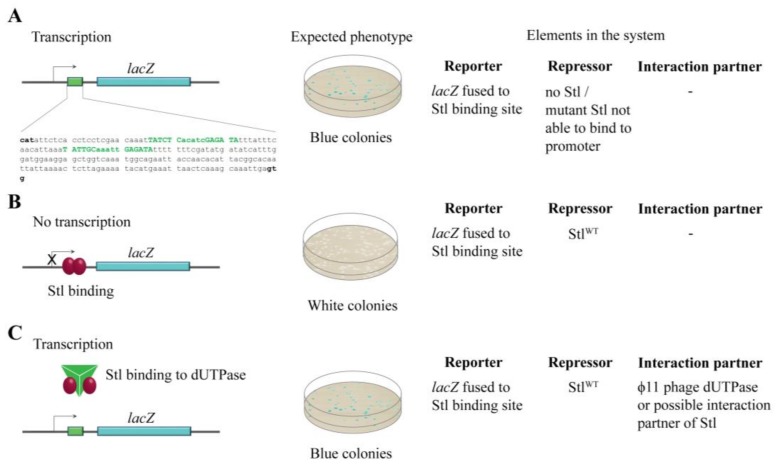
The design of the switch system. (**A**) In the absence of Stl-binding to its recognition sequence, *lacZ* can be expressed in the cell, leading to blue colonies in the experimental setup. The exact sequence from the SaPI_bov1_ genome (13733–13933) cloned into the *lacZ* promoter is shown. Specific Stl binding sites are labeled with green, conserved sites are shown with capital letters; (**B**) Stl binding to the promoter inhibits *lacZ* expression, leading to white colonies in the experimental setup; (**C**) The phage dUTPase protein sequestrates Stl^WT^ from the promoter DNA segment, leading to blue colonies.

The SaPI_bov1_ master repressor, Stl, has separate domains for DNA and protein binding [8]. The DNA binding function of Stl is performed through a helix-turn-helix (HTH) motif [8,9], as in case of the λcI repressor protein of the lambda phage [10,11]. A similar domain architecture was identified in other representatives of the family of Stl-like repressors encoded in different SaPIs as well [8]. These repressor proteins are highly diverse considering their amino acid sequence and higher order structure; however, there is considerable sequence conservation within their HTH segments. Using in silico prediction tools, the HTH motif could be found in eight out of the 12 investigated master repressors within the diverse *S*. *aureus* pathogenicity islands. In these repressors, the HTH motif was invariably located at the *N*-terminal part, similar to Stl. The *C*-terminal domain of the SaPI_bov1_ Stl repressor was proposed to contribute to the binding of protein interaction partner(s) [8]. The specific DNA segment recognized by the HTH motif of Stl was also recently characterized in detail [3] (cf. Figure 1 DNA sequence indicated in green). It was shown that Stl binds to a palindromic sequence, constituted by two 6-mer repeat sequences interrupted with a five-nucleotide long non-specific segment. Such palindromes can be found at the *stl-str* and the *str-xis* intergenic regions of the *S. aureus* genome, which is the regulation site of Stl controlling the Str and Xis expression [2,3]. 

The identification of the cognate DNA binding segments for the Stl repressor offers a basis for the design of a molecular switch. Switchable systems based on e.g., bacterial operons, transcription factors, or repressor proteins have been bioengineered for decades and are used for basic research as well as for various biotechnological applications. One of the first widely used systems for gene expression control in bacteria is the lactose repressor system (the *lac* operon) described by Jacob and Monod in 1961 [12]. Another extensively used switch-system for the regulation of recombinant protein production relies on the Tet repressor (TetR) [13,14] that drives the transcription of a family of tetracycline (tc) resistance determinants in Gram-negative bacteria. TetR can be used for the selective control of the expression of single genes in some organisms (plants and lower eukaryotes) without further modifications [14]. The several other commonly used inducible promoters include P_BAD_ [15], P_tac_ [16], P_trc_ [17], and P_T7_ [18]. Besides these, popular two-hybrid reporter systems may also apply transcription factors. In the bacterial two-hybrid system, one of the target proteins can be fused to the dimeric bacteriophage λcI repressor [19].

Our aim was to design and implement a switchable system in which the macromolecular interactions between Stl and its cognate DNA binding segments can be revealed. Despite our numerous trials, neither the flexible Stl protein on its own nor its complex with DNA could be crystallized. Hence, we needed another experimental approach to gain insights into the Stl:DNA interaction. Using the Stl-based reporter system, the molecular components responsible for Stl binding to DNA can be characterized in a high-throughput manner. We used *Mycobacterium smegmatis* (*M. smegmatis*) as the cellular host system for our studies, as Stl expression in mycobacterial cells is already implemented [20] and the large evolutionary distance between mycobacteria and *S. aureus* (Actinobacteria vs. Firmicutes, respectively) provides an opportunity to investigate the macromolecular interactions of Stl without putative perturbing effects. Finally, we extended our test system to investigate the Stl interaction with a phage protein, Φ11 dUTPase.

## 2. Materials and Methods

### 2.1. Bacterial Strains, Media, and Growth Conditions

The *E. coli* XL1-Blue and BL21 Rosetta strains were used for cloning and in vitro protein expression, respectively. The *M. smegmatis* mc^2^155 strain used for further experiments was grown in Lemco liquid culture or on solid plates with the addition of 15 g L^−1^ Bacto agar as described previously [21]. Kanamycin was added at 20 μg/mL, hygromycin B at 50 μg/mL, and chloramphenicol at 34 μg/mL. X-gal (5-bromo-4-chloro-3-indolyl-β-d-galactopyranoside) was used at 40 μg/mL, and tetracycline at 20 ng/mL for the induction of protein expression in *M. smegmatis*.

### 2.2. Cloning and Mutagenesis

For the construction of the p2NIL-LacZ^Str^-INT vector, the Stl-regulated promoter region [3] was PCR-amplified and cloned into the p2NIL vector using SalI and HindIII restriction sites. The *lacZ* gene was PCR-amplified from pGOAL17 [22] and cloned upstream of the promoter containing Stl binding sites using BglII and SalI restriction sites to generate the p2NIL-LacZ^Str^ vector. The integration cassette from the pUC-Gm-Int plasmid [23] was PCR amplified and cloned into the p2NIL-LacZ^Str^ plasmid using NotI restriction site to generate the p2NIL-LacZ^Str^-INT construct.

The pKW08-Stl^C-term^ and the pKW08-Stl^AA^ were generated by inserting the truncated or mutant Stl sequence amplified from pGEX-4T-1-Stl-CTD and pGEX-4T-1-Stl^AA^, respectively [8], into the pKW08-Lx vector using HindIII and BamHI restriction sites.

The colony PCR product of the random mutagenesis experiment was inserted into the pKW08 vector using BamHI and HindIII restriction sites to generate the pKW08-Stl^A236T^ plasmid.

The pGex-4T-1-Stl^A236T^ plasmid was generated by site-directed mutagenesis (QuikChange method, Stratagene) [24].

The pSJ27-ϕdut plasmid was constructed from the pSJ27 plasmid (kind gift of Graham F. Hatfull [25]) using recombination cloning. The ϕ11 dut gene sequence was synthesized by GenScript.

Primers used in this study are summarized in Supplementary Table S2. Successful cloning and mutagenesis was verified in all cases by sequencing of the appropriate region of the plasmid. All of the plasmids used in this study are summarized in Supplementary Table S3.

### 2.3. Construction of the M. smegmatis Reporter Strain

p2NIL-LacZ^Str^-INT plasmid (0.5 µg) was electroporated into electrocompetent wild-type *M. smegmatis* to generate the LacZ^Str^-carrying *M. smegmatis* strain. For inducible Stl expression, 0.5 µg of pKW08-Stl generated in Reference [20] was electroporated into electrocompetent LacZ^Str^ carrying *M. smegmatis* strain. Protein expression was verified by Western blot as previously described [20].

### 2.4. Analysis of Different Stl Mutants in the Reporter System

pKW08-Stl, pKW08-Stl^C-term^, pKW08-Stl^AA^, pKW08-Stl^A236T^ or pKW08-Stl^MUT^ (0.5 µg) was electroporated into the electrocompetent LacZ^Str^-carrying *M. smegmatis* strain. Then, 100 µL of the transformants was parallelly plated on kanamycin, hygromycin B, and X-gal or on kanamycin, hygromycin, X-gal, and tetracycline containing agar plates and incubated for 5 days at 37 °C. Blue colonies indicate *lacZ* transcription (no repression of the promoter), while white colonies indicate the functional repression of the promoter.

### 2.5. Random Mutagenesis of Stl

Random mutagenesis events were elicited using error-prone PCR conditions [26] comprising am extra 6 mM MgCl_2_ and 0.5 mM MnCl_2_, resulting in less faithful replication by the RedTaq polymerase (Sigma, St. Louis, MO, USA). The mutagenized product was cloned in pKW08-Lx using BamHI and HindIII restriction sites generating the pKW08-Stl^MUT^ library. The pKW08-Stl^MUT^ library was then electroporated into the LacZ^Str^-carrying *M. smegmatis* strain reviewed in the previous paragraph. The inserted *stl* sequence was amplified by colony PCR from selected blue colonies and subjected to sequencing. Primers used for cloning, PCR, and sequencing are compiled in Supplementary Table S2.

### 2.6. Protein Expression and Purification

The *E. coli* strain BL21 Rosetta (DE3) was transformed with the pGex-4T1-Stl and pGex-4T-1-Stl^A236T^ vectors and propagated in 500 ml Luria-Bertani broth (LB) to an OD_600_ of 0.6. The culture was then cooled to 30 °C and induced with 0.5 mM iso-propyl-β-d-thiogalactoside for 4 h. Subsequent manipulation of the harvested cells was carried out on ice. For Stl purification, cell pellets were solubilized in 30 mL of lysis buffer (50 mM HEPES (pH 7.5), 200 mM NaCl) supplemented with 2 mM dithiothreitol (DTT), 2 μg/mL RNase and DNase, and ethylenediaminetetraacetic acid (EDTA) free protease inhibitor (cOmplete ULTRA Tablets, Mini). Cell suspensions were sonicated (60 s, four times) and centrifuged (16,000× *g* for 30 min). Supernatant was loaded on a glutathione-agarose affinity-chromatography column. The column was washed with 10 volumes of lysis buffer. On-column cleavage was then performed by the addition of 80 cleavage unit thrombin to remove the glutathione-S-transferase tag. Following overnight cleavage, Stl was eluted from the column at >95% purity, as estimated by sodium dodecyl sulfate polyacrylamide gel electrophoresis (SDS-PAGE).

### 2.7. Electrophoretic Mobility Shift Assay (EMSA)

EMSA experiments were performed using a 229-mer dsDNA oligonucleotide of the Stl regulated promoter region based on our previous results [3] (cf. Supplementary Table S2). Complementary oligonucleotides were custom synthesized by Eurofins MWG Operon and hybridized by controlled gradual cooling after 5 min of incubation at 95 °C. The investigated proteins were mixed with 100 ng DNA in 20 μL total volume. After incubation for 5 min at room temperature, samples were loaded onto 8% polyacrylamide gel. Pre-electrophoresis was performed for 1 h at 150 V in tris-borate-EDTA (TBE) buffer, followed by sample electrophoresis for 70 min at room temperature at 100 V. Bands were detected using GelRed (Biotium, Fremont, CA, USA) and a Bio-Rad gel documentation system. The EMSA experiment was repeated three times. 

### 2.8. Construction of the Double Switch System to Test Protein:Protein Interaction

For the construction of the Stl:ϕ11 dUTPase double switch system, pSJ27-ϕdut vector was electroporated into the Stl- and LacZ^Str^-carrying *M. smegmatis* strain.

## 3. Results

### 3.1. Design and Construction of a Reporter System to Characterize Different Functions of the Stl Protein

The exact DNA sequence of the cognate Stl recognition site within the SaPI promoter region has already been determined [3]. Based on this knowledge, we constructed a switchable gene expression system using the Stl:DNA interaction.

To explore the Stl:DNA interaction in the cellular environment, the reporter system shown in Figure 1 was constructed in *M. smegmatis*. The reporter system is based on the widely used blue-white colony selection strategy, i.e., the activity of a β-galactosidase reporter gene (*lacZ*) leads to the conversion of the substrate X-gal to a blue-colored product providing a straightforward readout. We fused the *lacZ* gene to the specific Stl binding sequence [3,27] so that the Stl:DNA interaction inhibits *lacZ* transcription (white colonies). In the absence of Stl or when Stl:DNA binding is compromised, i.e., when either a mutant Stl or a phage protein able to sequestrate Stl is present, *lacZ* is expressed, leading to blue colonies (Figure 1).

**Figure 2:**
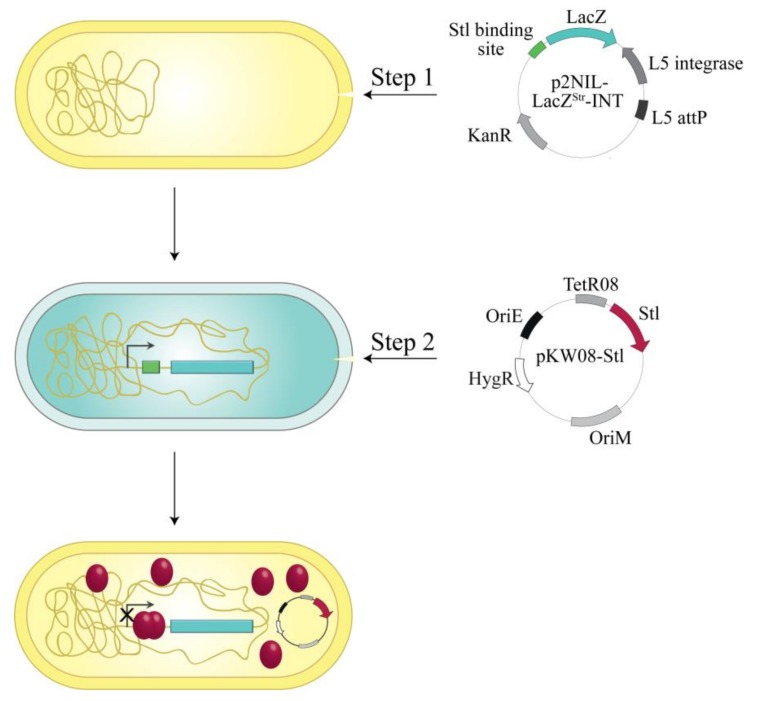
Workflow of the in vivo Stl:DNA binding assay: The p2NIL-LacZ^Str^-int vector carrying the desired Stl binding site in the promoter region of *lacZ* gene was electroporated into *M. smegmatis* cells (Step 1). After electroporation, this vector integrates into the genome. This is followed by Step 2: electroporation of pKW08-Stl vector into the Stl switch system. Stl expression is inducible in a tetracycline-dependent way.

The reporter plasmid also contained a L5 integration cassette [23] enabling it to integrate into the mycobacterial genome to the specific attP site (Figure 2). As a result of stable integration, transformed mycobacterium cells possess only one copy of the reporter system and therefore the color intensity of the β-galactosidase reaction is not dependent on the copy number of the vector. For inducible Stl expression, the cells containing the reporter construct were transformed with the plasmid pKW08-Stl (generated in Reference [20]).

### 3.2. Validation of the Switch System Using Wild-Type and Mutant Stl Constructs

**Figure 3:**
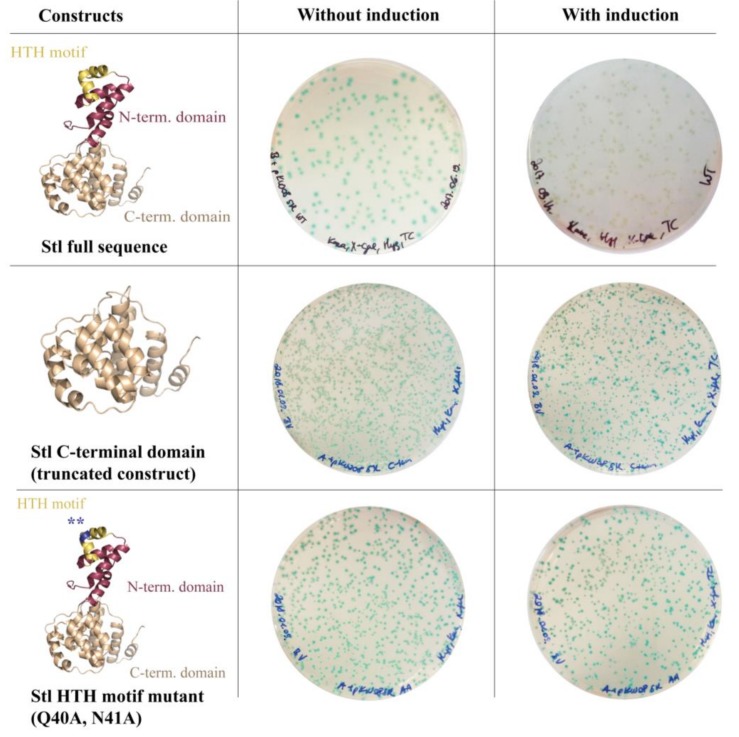
Validation of the switch system: To test the system functionality, several Stl constructs with various DNA-binding abilities were used. Without induction, i.e., in the absence of the Stl expression inducer tetracycline, cells form blue colonies in all cases. Upon the induction of the full-length wild-type Stl, white colonies are formed as Stl is able to fully repress the *lacZ* gene. Both the truncated Stl *C*-terminal domain construct and the HTH motif mutant Stl have diminished DNA-binding ability [8] and, as expected, their expression in *M. smegmatis* leads to the formation of blue colonies. Note that the difference in the number of colonies between variants represents the transformation efficiency.

To test the system functionality, several Stl constructs with various DNA-binding abilities were expressed. Full-length (i.e., wild-type) Stl was used as positive control. As described previously, both specific truncation and rationally designed point-mutations within the Stl protein sequence could lead to a significant reduction of the DNA-binding capability [8]. The Stl^C-term^ truncated construct is practically unable to bind to DNA since it lacks the N-terminus of the protein encoding the HTH motif, the bona fide segment for DNA binding. The mutant Stl^AA^ containing mutations within the HTH motif was shown to be significantly perturbed in DNA-binding as well [8]. The results of the validation experiment are shown in Figure 3. The Stl protein is presented as a molecular model in the figure. This model was constructed based on vacuum circular dichroism (CD) experiments and model building [8]. Blue colonies formed in all cases without the induction of Stl expression. Upon the induction of Stl expression, white colonies formed with the full-length wild-type Stl as it is able to fully repress the transcription of the *lacZ* gene (colony counting revealed that 70% of the colonies were white). The expression of both the Stl^C-term^ and Stl^AA^, having diminished DNA-binding ability in vitro, leads to the formation of blue *M. smegmatis* colonies, confirming that the system is functional (colony counting data: 100% blue colonies, i.e., no white colonies were observed). 

### 3.3. Random Mutagenesis-Based Search for Stl Mutations Affecting DNA-Binding

**Figure 4:**
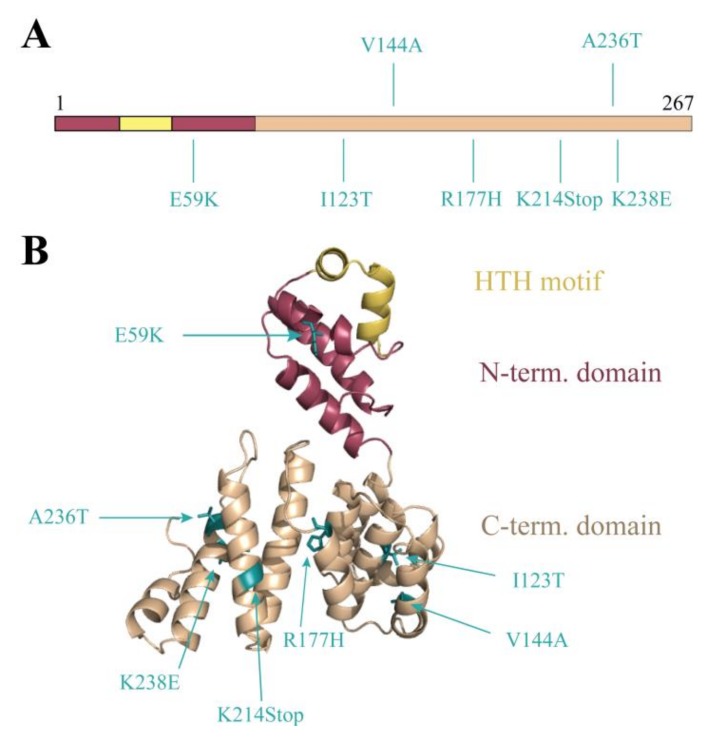
Probing DNA binding by random mutagenesis: Random mutagenesis PCR was carried out on the Stl coding sequence generating various single mutations. The PCR product was cloned into pKW08 vector and electroporated into the Stl switch system. The depicted mutants showed blue phenotype in the designed Stl switch system, suggesting defective DNA-binding ability. (**A**) The location of the hits in the Stl full-length protein; (**B**) Mutants mapped on the Phyre2 structural model of Stl [8].

We wished to apply this switch system as a non-biased high-throughput approach to characterize the Stl:DNA interaction with a focus on the protein residues that are involved in DNA-binding. The Stl was therefore subjected to random mutagenesis. The random mutagenesis PCR reaction mix contained excess Mg^2+^ and Mn^2+^ ions for a less faithful replication of the DNA by the polymerase [26]. The mutagenized product was cloned and electroporated into mycobacteria hosting the LacZ^Str^ reporter system. Blue colonies indicating the lack of *lacZ* transcription were selected. These blue colonies represented inefficient Stl:DNA interaction. The Stl coding sequence was therefore amplified by colony PCR and sent to sequencing. A wide range of mutations were identified (cf. Supplementary Table S1 for a full list). Figure 4A focuses on the several mutations occurring as single mutation events in the Stl sequence (Stl^E59K^, Stl^I123T^, Stl^V144A^, Stl^R177H^, Stl^K214Stop^, Stl^A236T^, Stl^K238E^). We projected these mutations upon the previously published three-dimensional (3D) structural model of Stl [8] (Figure 4B). The analysis of the 3D structural model revealed plausible causes for the perturbing effects for several of the listed mutations. For example, the E59K mutation that results in changing the negatively charged glutamate to a positively charged lysine in the vicinity of the HTH motif presumably alters the electrostatic potential and hence may perturb DNA binding. Two of the mutations, I123T and R177H, lead to steric clashes in the model suggesting a strongly perturbed protein structure. The effect of the V144A mutation is not straightforwardly explained by in silico data only. The K214Stop mutant may be too short to be functional. The K238E again represents a large change in the charge of the replaced side chain. The hydrophobic to polar change in the A236T mutant may have impact on intramolecular interactions that contribute to the flexibility of the 3D structure, although the functional effect of this mutation predicted in the structural model is minor compared to most of the other mutations. We chose to further investigate this hit both in vivo and in vitro in order to verify that the random mutagenesis approach resulted in valid hits. This is indicative of the sensitivity of our screening system if even this minor structural change represents a valid hit.

### 3.4. Analysis of the Stl^A236T^ DNA Binding Properties In Vivo and In Vitro

**Figure 5:**
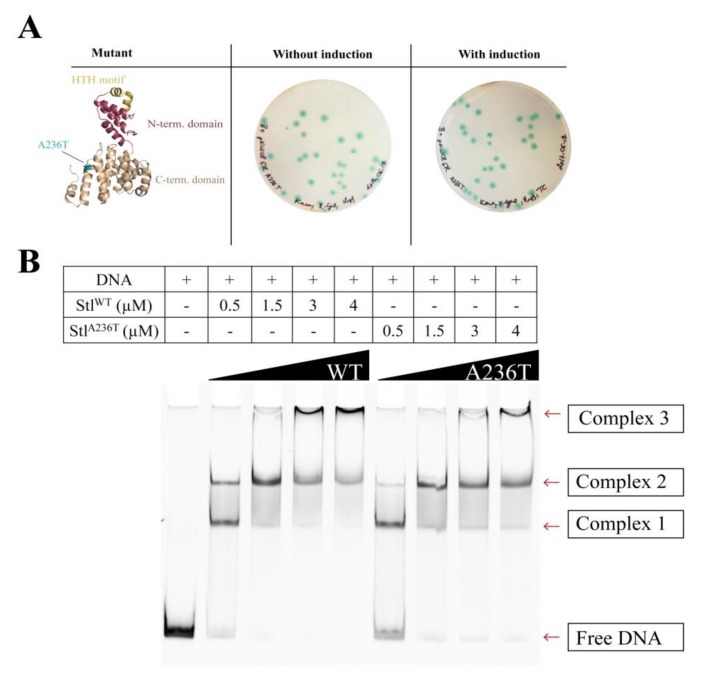

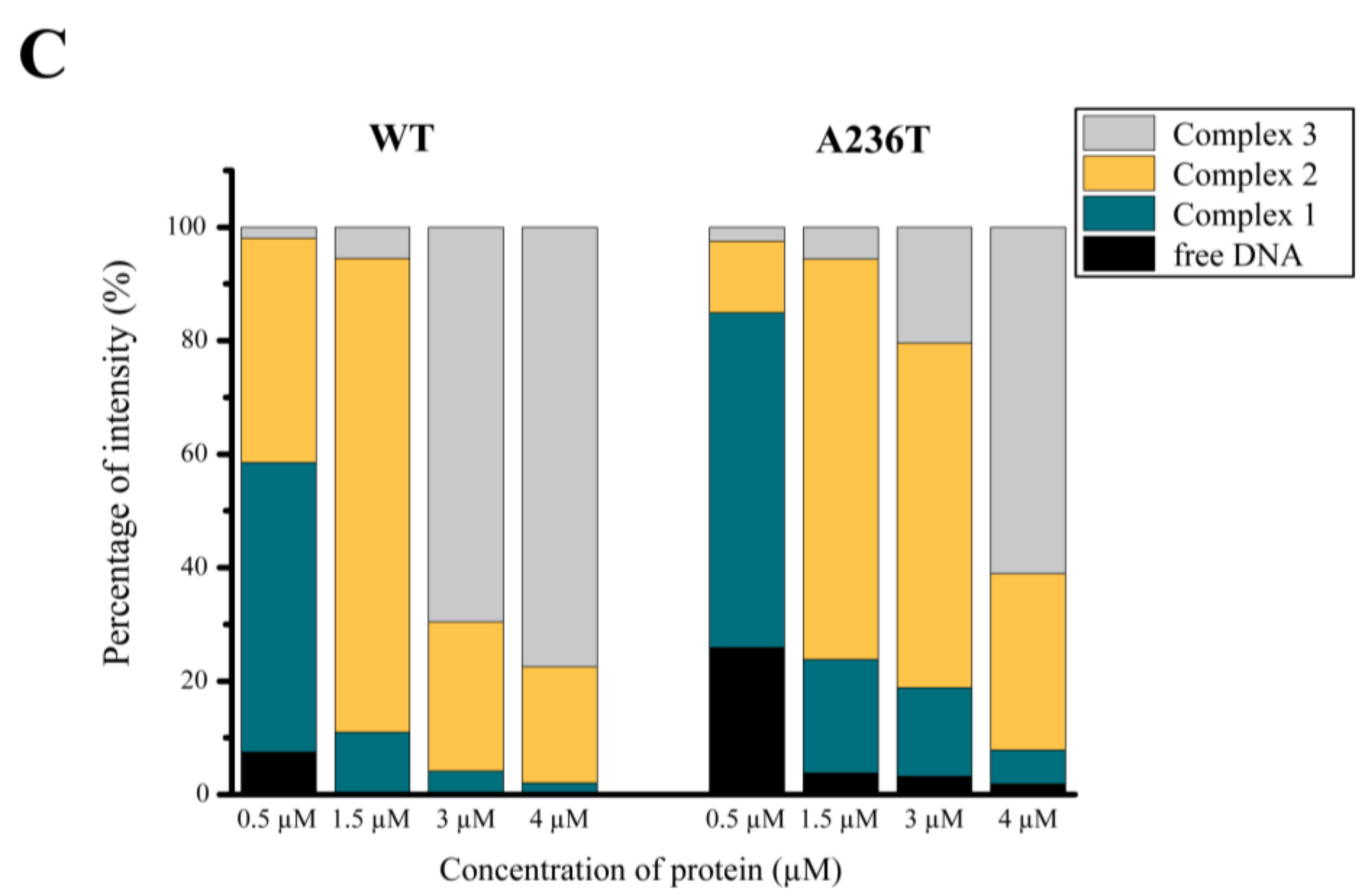
Characterization of the Stl^A236T^ mutant (**A**) Testing the Stl^A236T^ mutant in the Stl switch system. The structural model locates the A236T mutation at the *C*-terminal domain shown in blue. The HTH motif is colored yellow and the *N*-terminal domain is colored maroon. The expression of the Stl^A236T^ mutant resulted in blue colonies, verifying that it is defective in DNA binding in vivo; (**B**) Comparative electrophoretic mobility shift assay (EMSA) with Stl^WT^ and Stl^A236T^. Stl^A236T^ is less prone to complex formation with DNA. Therefore, more of the free DNA is visible at the corresponding bands than that of Stl^WT^. The comparative EMSA was repeated three times with same results; (**C**) Densitometry results of each of the species exhibiting different electrophoretic mobilities are shown by colored bands. The discrepancy between the wild-type and mutant Stl is quite noticeable, confirming our previous in vivo results. These data suggest that the DNA binding of Stl^A236T^ is compromised in vitro.

To exclude any background effect in the previous random mutagenesis experiment, we cloned and expressed the Stl^A236T^ mutant in the Stl switch system (Figure 5A). The exclusive appearance of blue colonies clearly showed that the mutation disrupted the DNA binding ability of Stl in the cellular environment. As an in vitro test for DNA binding, we carried out EMSA using purified proteins and a 229-mer dsDNA oligonucleotide of the Stl-regulated promoter region (Figure 5B shows a representative experiment). It is clearly seen in the gel that the band corresponding to free DNA disappears in conjunction with the appearance of various DNA-protein complexes upon the increase of the protein concentration (Figure 5B). The densitometric analysis of the EMSA gel shows that the free DNA disappears at 1.5 µM Stl concentration when wild-type Stl is added, while free DNA is still present at even 4 µM concentration of the Stl^A236T^ mutant (Figure 5C). This indicates the diminished DNA binding capability of the Stl^A236T^ mutant compared to the wild-type Stl. The fact that the Stl^A236T^ does not lose its DNA binding capability completely in the EMSA indicates that its overall structure is probably not affected by the mutation. This result also suggests that a relatively minor difference in the structure and a moderate decrease in the in vitro DNA binding affinity could explain the loss of in vivo DNA binding.

Both the in vivo and in vitro validation experiments using the Stl^A236T^ mutant demonstrated that our Stl switch system is suitable for the identification of protein segments responsible for DNA binding.

### 3.5. The Investigation of Protein:Protein Complexation Using the LacZ^Str^ Reporter System

**Figure 6:**
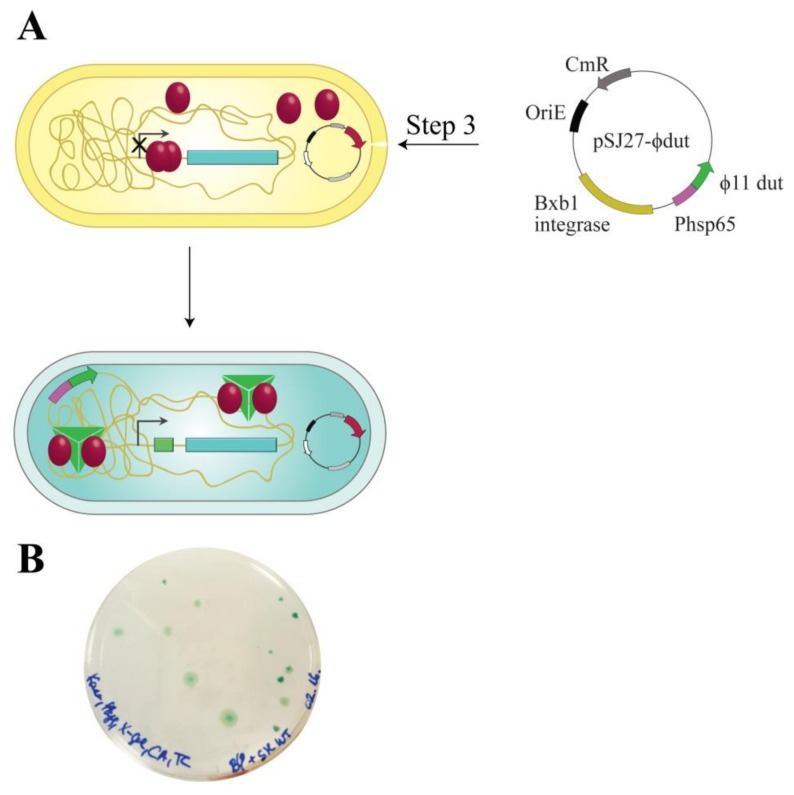
Workflow of the double switch system. (**A**) pSJ27-Φ11dut vector was electroporated into the Stl switch system. The relatively high expression level of Φ11 phage dUTPase enzyme is provided by a heat shock protein promoter (Phsp65). Φ11 phage dUTPase is a known partner of Stl, able to promote Stl to release the regulated DNA [24,28]; (**B**) Validation of the detection of protein:protein interaction using the LacZ^Str^ reporter system. Stl in complex with Φ11 phage dUTPase releases DNA, therefore, blue colonies are formed. Note that the number of colonies represents the transformation efficiency.

As it is known from the literature, complexation between Stl and different phage proteins has immediate significance in spreading virulence factors. The LacZ^Str^ reporter system carries the opportunity to test the interaction of Stl with phage proteins that remove Stl from its cognate DNA binding site. We probed a known interaction with the ϕ11 phage dUTPase to test this hypothesis [24,28]. Figure 6A shows the workflow of the double switch experiment. ϕ11 phage dUTPase expression was tuned up via a strong expression promoter, Phsp65, to ensure dUTPase excess relative to Stl. As a result, we observed 100% of blue colonies (Figure 6B), indicating that Stl was effectively sequestrated from the lacZ promoter. This experiment confirms that the switch system is applicable to the detection of protein:protein interactions. This feature also presents the opportunity to test phage protein libraries in search for new derepressing interactions.

## 4. Discussion

Switchable systems in molecular biology are associated with well-defined advantages and far-reaching applications in medical and industrial biotechnologies. These systems are based on cognate molecules that turn on/off metabolic pathways, signal transduction processes, and gene expression regulation. The actual benefit for each of these systems is defined by their specificity and applicability in various cellular models and/or organisms. In the present study, our aim was to exploit the phage-bacterium interaction and to design a molecular switch using the components regulating how phages modulate bacterial cell physiology.

The setup of our system is similar to that of the bacterial one-hybrid (B1H) system [29]; however, the two systems differ in their applicability and the actual molecular components. The B1H system was designed to identify target sites of a given DNA binding domain, while our system is constructed to identify protein segments crucial for DNA binding. In the B1H system, DNA binding activates the transcription of the reporter genes, whereas in the Stl-driven switch system, DNA binding blocks the transcription of the reporter gene. In addition, the transcription factor is expressed as a fusion to a subunit of RNA polymerase in the case of the B1H system, while the Stl repressor is able to bind and repress the promoter of the reporter gene at the same time and does not therefore need to be fused to another protein.

Our system has unique advantages in investigating protein:DNA interactions. First, the DNA binding segments of the protein can be identified in a single round of in vitro selection. Therefore, no additional protein purification steps or antibodies are required. Additionally, our method can potentially be used to discover phage-host protein:protein interactions by screening large DNA libraries. In our approach we exploited the capability of the Stl repressor to bind to specific DNA segments in a genetically engineered *M. smegmatis* strain that contained the marker β-galactosidase gene (*lacZ*) and the Stl-specific DNA binding segments in close proximity on the bacterial chromosome. Due to the integration vector relying on attP sites, both the *lacZ* gene and the Stl-specific DNA segment are present in just a single copy. Thus, the signal generated in this engineered system is stable among all cells present in the culture and there is no copy-number variation. 

In contrast, Stl is expressed from an episomal vector. As an advantage, the amino acid sequence of the Stl repressor can be optionally modified, e.g., by random mutagenesis, and high variety of mutant repressors can be investigated in a high-throughput manner. In addition, protein expression from this vector is inducible, allowing efficient control of the expression of wild-type or mutated Stl proteins [20].

The application of the molecular switch system in the engineered *M. smegmatis* strain enabled us to focus on the Stl:DNA interaction in an unbiased manner using a random-mutagenized Stl library. Detailed characterization of protein:DNA interaction is of key current interest, with an additional biomedical aspect in our case, as we investigate the Stl protein involved in the regulation of pathogenicity factors in *S. aureus*. The virulence of *S. aureus* depends on helper phages which promote SaPI derepression by sequestering Stl. Interestingly, it is the phage dUTPase enzyme that interacts with Stl and conveys the derepression effect [4]. For the superfamily of dUTPases, Stl is the first well-established protein interacting partner; however, previous observations also suggested protein:protein interactions for dUTPases from other species [30,31]. 

In our engineered *M. smegmatis* strain, we exploit the β-Gal reporter system associated with a straightforward, easy readout suitable for high-throughput studies. This reporter system derived from the phage-bacterium interaction can be used to map DNA-binding protein surfaces in the living cell.

In this study, we demonstrated that the Stl switch system is capable of identifying side chain mutations in the Stl protein that are functionally involved in the Stl:DNA macromolecular complex formation. The random mutagenesis-generated mutations were projected on the 3D structural model of Stl. Interestingly, we observed that several residues in the *C*-terminal domain may also contribute to Stl DNA binding in the context of the full-length protein (cf. [8]). For some of these mutations, the actual phenotype was simple to rationalize due to major changes in the character of the residue involved in the mutation. We selected one specific mutant and subjected it to detailed in vivo and in vitro characterization. Results confirmed that this hit was fully valid. Hence, the herein presented system is capable of providing novel insights into macromolecular interactions involving Stl. As discussed earlier, Stl is an important repressor in the lifecycle of Staphylococcal pathogenicity islands and Staphylococcal phages [4]. Our test system is based on the perturbation of the Stl-induced switch in gene expression control. As such, our test system is also amenable to further biotechnological applications focusing on the identification of phage proteins that may perturb Stl-function. We have presented one such example for the Stl-interacting phage dUTPase protein. Phage libraries can also be tested for Stl-perturbing function in our switch system, where phage proteins can be investigated for their effect in a systematic approach. Previously, similar approaches were performed within the Staphylococcal cell and as such the possibility of identification of those proteins that may perturb Stl function and are essential for the phage lifecycle was practically excluded [4,7]. Importantly, this limitation is overcome in our system, which operates in the *M. smegmatis* environment and allows the investigation of complete phage libraries.

## Supplementary Materials

The following are available online at http://www.mdpi.com/1999-4915/10/4/168/s1, Table S1: Stl mutants identified with reduced DNA binding ability in the Stl switch system, Table S2: Oligonucleotides used in the present study, Table S3: Plasmids used in the present study.
