# Supplementary material for: Exploiting a Phage-Bacterium Interaction System as a Molecular Switch to Decipher Macromolecular Interactions in the Living Cell
Source: Viruses. 2018 Apr 1;10(4):168. doi: 10.3390/v10040168 (PMC5923462; doi:10.3390/v10040168)
Supplement: Supplementary file 1 [file viruses-10-00168-s001.zip › Supplementary_Materials_Suranyi_Viruses/SUPPLEMENTARY MATERIALS_2018_03_22.docx]

**Supplementary Materials to accompany the manuscript:**

Exploiting a phage-bacterium interaction system as a molecular switch to decipher macromolecular interactions in the living cell

Éva Viola Surányi ^1,2,†,^* , Rita Hirmondó ^2,†,^*, Kinga Nyíri ^1,2^, Szilvia Tarjányi ^2^, Bianka Kőhegyi ^1,2^, Judit Tóth ^2^ and Beáta G. Vértessy ^1,2,^*

^1^ Department of Applied Biotechnology and Food Sciences, Budapest University of Technology and Economics, Budapest, H-1111, Hungary

^2^ Institute of Enzymology, RCNS, Hungarian Academy of Sciences, Budapest, H-1117, Hungary

† These authors contributed equally to this work as first authors

***** Correspondence: vertessy@mail.bme.hu, vertessy.beata@ttk.mta.hu; Tel.: +36 13 826 707, eva.suranyi@mail.bme.hu; Tel.: +36 13 826 729, [hirmondo.rita@ttk.mta.hu](mailto:hirmondo.rita@ttk.mta.hu); Tel.: +36 13 826 729

**SUPPLEMENTARY MATERIALS**

**Supplementary Table S1.** Stl mutants identified with reduced DNA binding ability in the Stl switch system

| **Number of mutations** | **Stl mutants** |
| --- | --- |
| 1 mutation | Stl^K19K^ |
|  | Stl^E59K^ |
|  | Stl^G62Afs*88^ |
|  | Stl^G66Afs*88^ |
|  | Stl^I123T^ |
|  | Stl^Y143Y^ |
|  | Stl^V144A^ |
|  | Stl^R177H^ |
|  | Stl^K214*^ |
|  | Stl^A236T^ |
|  | Stl^K238E^ |
| 2 mutations | Stl^Q6H, S76T^ |
|  | Stl^G15S, K240R^ |
|  | Stl^T16S, D142A^ |
|  | Stl^G92D, V229M^ |
|  | Stl^I134V, H256R^ |
|  | Stl^K158N, I220V^ |
| 3 mutations | Stl^D174V, G185G, K193K^ |
| 4 mutations | Stl^I18T, Y98H, D142D, R227C^ |
|  | Stl^V55M, F79L, N137D, I161T^ |
| 5 mutations | Stl^Y70C, K80K, K93R, L152R, I181Lfs*188^ |
| 6 mutations | Stl^M1Rª, G54V, L129P, D140E, E186V, H188H^ |
|  | Stl^I17V, F38S, L72P, G92S, D95G, D108N^ |
|  | Stl^N41D, E186V, R227R, D235D, K240R, K244E^ |
| 7 mutations | Stl^I17T, S76S, G92A, I212T, L222P, R227R , I237I^ |
| 8 mutations | Stl^S30G, Y84H, I134A, Y143H, N168S, L194L, E224E, Q257Q^ |
| 10 mutations | Stl^F38L, H46Y, N48S, I58T, L65P, P86Q, Y112H, S114N, N135I, N203S^ |
| 11 mutations | Stl^K31N, I53I, K63K, I67I, R74H, K93K, D155D, K193E, T197A, L245P, Y246*^ |

ªpKW08-Stl vector is containing an AU-tag before the coding sequence of Stl (Hirmondó et al. 2015, DNA Repair), therefore a protein may be translated from this mutant despite the first Met is mutated

**Supplementary Table S2. Oligonucleotides used in the present study**

Restriction sites are underlined

| **Used in** | **Oligo name** | **5’-3’ sequence** |
| --- | --- | --- |
| **Cloning of p2NIL-LacZ^Str^-INT plasmid** | SalI_str | TTTTAGTCGACCATATTCTCACCTCCTCGAAC |
|  | HindIII_str | TGTGTAAGCTTCATATTCTCACCTCCTCGAAC |
|  | BglII_LacZ | TGTGTAGATCTGTCGTTGTGGTCACTCG |
|  | SalI_LacZ | TATATGTCGACCGCCCAAACATGCATGGAT |
|  | NotI_INT_for | ATATAGCGGCCGCTGCTCCATAACATCAAACATC |
|  | NotI_INT_rev | ATATAGCGGCCGCGAAGCTTGCATGCCTGC |
| **Cloning of pKW08-Stl^C-term^** | Stl-Cterm_Au_BamHI_f | ATTAGGATCCATGGATACGTATCGCTACATAAGCCCGACCCTGAACG |
|  | Stl_HindIII_r | ATTAAAAGCTTGCGGCCGCTTAGTTGGTATC |
| **Error prone PCR, Cloning of pKW08-Stl^A236T^**  **and pKW08-Stl^MUT^, and pKW08-Stl^AA^** | Stl_Au_BamHI_f | AATTAGGATCCATGGATACGTATCGCTACATAGCTAGCC |
|  | Stl_HindIII_r | ATTAAAAGCTTGCGGCCGCTTAGTTGGTATC |
| **Colony PCR for sequencing** | Stl_seq_f | GGTGGTGAGTCATAGTTGC |
|  | Stl_seq_r | CGCTTAATCCAAAGTTCAAACG |
| **EMSA** | Stl-Str | TCGTAAACATATTCTCACCTCCTCGAACAAATTATCTCACATCGAGATATTTATTTCAACATTA  AATATTGCAAATTGAGATATTTTTTTCGATATGATATCATTTGGATGGAAGGAGCTGGTCAAA  TGGCAGAATTACCAACACATTACGGCACAATTATTAAAACTCTTAGAAAATACATGAAATTAA  CTCAAAGCAAATTGAGTGAAAGGACAGGATTTAGGATCC |

**Supplementary Table S3. Plasmids used in the present study**

| **Plasmid name** | **Characteristics** | **Antibiotic Resistance** | **Reference** |
| --- | --- | --- | --- |
| p2NIL-LacZ^Str^-INT | reporter plasmid; LacZ; L5 integration cassette | Kan^R^ | this study |
| pKW08-Stl | expression of Stl in Mycobacterium; Tet-inducible | Hyg^R^ | Hirmondo et al., 2015 |
| pKW08-Stl^C-term^ | expression of C-terminal part of Stl in Mycobacterium; Tet-inducible | Hyg^R^ | this study |
| pKW08-Stl^AA^ | expression of AA mutant Stl in Mycobacterium; Tet-inducible | Hyg^R^ | this study |
| pKW08-Stl^MUT^ | expression of random mutagenized Stl in Mycobacterium; Tet-inducible | Hyg^R^ | this study |
| pKW08-Stl^A236T^ | expression of A236T mutant Stl in Mycobacterium; Tet-inducible | Hyg^R^ | this study |
| pGex-4T-1-Stl | protein expression of Stl, Gluthation-S-transferase tag | CA^R^ | Nyiri et al., 2015 |
| pGex-4T-1-Stl^A236T^ | protein expression of A236T mutant Stl, Gluthation-S-transferase tag | CA^R^ | this study |
| pSJ27-ϕdut | expression of ϕ11 dUTPase in Mycobacterium; Bxb1 integration cassette | Cm^R^ | this study |
