# Supplementary material for: Exploiting a Phage-Bacterium Interaction System as a Molecular Switch to Decipher Macromolecular Interactions in the Living Cell
Source: Viruses. 2018 Apr 1;10(4):168. doi: 10.3390/v10040168 (PMC5923462; doi:10.3390/v10040168)
Supplement: Supplementary file 1 [file viruses-10-00168-s001.zip › Supplementary_Materials_Suranyi_Viruses/SUPPLEMENTARY MATERIALS_2018_03_22.pdf]

Supplementary Materials to accompany the manuscript:

Exploiting a phage-bacterium interaction system as a molecular switch to decipher macromolecular interactions in the living cell

Éva Viola Surányi [1,2,†,*] , Rita Hirmondó [2,†,*], Kinga Nyíri [1,2], Szilvia Tarjányi [2], Bianka Kőhegyi [1,2], Judit Tóth [2] and Beáta G. Vértessy [1,2,*]

[1] Department of Applied Biotechnology and Food Sciences, Budapest University of Technology and Economics, Budapest, H-1111, Hungary

[2] Institute of Enzymology, RCNS, Hungarian Academy of Sciences, Budapest, H-1117, Hungary

† These authors contributed equally to this work as first authors

* Correspondence: vertessy@mail.bme.hu, vertessy.beata@ttk.mta.hu; Tel.: +36 13 826 707, eva.suranyi@mail.bme.hu; Tel.: +36 13 826 729, hirmondo.rita@ttk.mta.hu; Tel.: +36 13 826 729

SUPPLEMENTARY MATERIALS

Supplementary Table S1. Stl mutants identified with reduced DNA binding ability in the Stl switch system

**Number of mutations**	**Stl mutants**
1 mutation	Stl^{K19K}
	Stl^{E59K}
	Stl^{G62Afs*88}
	Stl^{G66Afs*88}
	Stl^{I123T}
	Stl^{Y143Y}
	Stl^{V144A}
	Stl^{R177H}
	Stl^{K214*}
	Stl^{A236T}
	Stl^{K238E}
2 mutations	Stl$^{Q6H, S76T}$
	Stl$^{G15S, K240R}$
	Stl$^{T16S, D142A}$
	Stl$^{G92D, V229M}$
	Stl$^{I134V, H256R}$
	Stl$^{K158N, I220V}$
3 mutations	Stl$^{D174V, G185G, K193K}$
4 mutations	Stl$^{I18T, Y98H, D142D, R227C}$

	Stl$^{\text{V55M, F79L, N137D, I161T}}$
5 mutations	Stl$^{\text{Y70C, K80K, K93R, L152R, I181Lfs*188}}$
6 mutations	Stl$^{\text{M1R}^{a}\text{, G54V, L129P, D140E, E186V, H188H}}$
	Stl$^{\text{I17V, F38S, L72P, G92S, D95G, D108N}}$
	Stl$^{\text{N41D, E186V, R227R, D235D, K240R, K244E}}$
7 mutations	Stl$^{\text{I17T, S76S, G92A, I212T, L222P, R227R , I237I}}$
8 mutations	Stl$^{\text{S30G, Y84H, I134A, Y143H, N168S, L194L, E224E, Q257Q}}$
10 mutations	Stl$^{\text{F38L, H46Y, N48S, I58T, L65P, P86Q, Y112H, S114N, N135I, N203S}}$
11 mutations	Stl$^{\text{K31N, I53I, K63K, I67I, R74H, K93K, D155D, K193E, T197A, L245P, Y246*}}$

[a]pKW08-Stl vector is containing an AU-tag before the coding sequence of Stl (Hirmondó et al. 2015, DNA Repair), therefore a protein may be translated from this mutant despite the first Met is mutated

Supplementary Table S2. Oligonucleotides used in the present study

Restriction sites are underlined

Used in	Oligo name	5’-3’ sequence
**Cloning of p2NIL-LacZ^{Str}-INT plasmid**	SalI_str	TTTTAGTCGACCATATTCTCACCTCCTCGAAC
	HindIII_str	TGTGTAAGCTTCATATTCTCACCTCCTCGAAC
	BglII_LacZ	TGTGTAGATCTGTCGTTGTGGTCACTCG
	SalI_LacZ	TATATGTCGACCGCCCAAACATGCATGGAT
	NotI_INT_for	ATATAGCGGCCGCTGCTCCATAACATCAAACATC
	NotI_INT_rev	ATATAGCGGCCGCGAAGCTTGCATGCCTGC
**Cloning of pKW08-Stl$^{C\text{-}term}$**	Stl-Cterm_Au_BamHI_f	ATTAGGATCCATGGATACGTATCGCTACATAAGCCCGACCCTGAACG
	Stl_HindIII_r	ATTAAAAGCTTGCGGCCGCTTAGTTGGTATC
**Error prone PCR, Cloning of pKW08-Stl^{A236T} and pKW08-Stl^{MUT}, and pKW08-Stl^{AA}**	Stl_Au_BamHI_f	AATTAGGATCCATGGATACGTATCGCTACATAGCTAGCC
	Stl_HindIII_r	ATTAAAAGCTTGCGGCCGCTTAGTTGGTATC
**Colony PCR for sequencing**	Stl_seq_f	GGTGGTGAGTCATAGTTGC
	Stl_seq_r	CGCTTAATCCAAAGTTCAAACG
**EMSA**	Stl-Str	TCGTAAACATATTCTCACCTCCTCGAACAAATTATCTCACATCGAGATATTTATTTCAACATTAAATATTGCAAATTGAGATATTTTTTTCGATATGATATCATTTGGATGGAAGGAGCTGGTCAAATGGCAGAATTACCAACACATTACGGCACAATTATTAAAACTCTTAGAAAATACATGAAATTAACTCAAAGCAAATTGAGTGAAAGGACAGGATTTAGGATCC

Supplementary Table S3. Plasmids used in the present study

Plasmid name	Characteristics	Antibiotic Resistance	Reference
p2NIL-LacZ^{Str}-INT	reporter plasmid; LacZ; L5 integration cassette	Kan^{R}	this study
pKW08-Stl	expression of Stl in Mycobacterium; Tet-inducible	Hyg^{R}	Hirmondo et al., 2015
pKW08-Stl$^{C\text{-}term}$	expression of C-terminal part of Stl in Mycobacterium; Tet-inducible	Hyg^{R}	this study
pKW08-Stl^{AA}	expression of AA mutant Stl in Mycobacterium; Tet-inducible	Hyg^{R}	this study
pKW08-Stl^{MUT}	expression of random mutagenized Stl in Mycobacterium; Tet-inducible	Hyg^{R}	this study
pKW08-Stl^{A236T}	expression of A236T mutant Stl in Mycobacterium; Tet-inducible	Hyg^{R}	this study
pGex-4T-1-Stl	protein expression of Stl, Gluthation-S-transferase tag	CA^{R}	Nyiri et al., 2015
pGex-4T-1-Stl^{A236T}	protein expression of A236T mutant Stl, Gluthation-S-transferase tag	CA^{R}	this study
pSJ27-ϕdut	expression of ϕ11 dUTPase in Mycobacterium; Bxb1 integration cassette	Cm^{R}	this study
